# Perturbations From Ducts on the Modes of Acoustic Thermometers

**DOI:** 10.6028/jres.114.019

**Published:** 2009-10-01

**Authors:** K. A. Gillis, H. Lin, M. R. Moldover

**Affiliations:** National Institute of Standards and Technology, Gaithersburg, MD 20899-0001

**Keywords:** acoustic resonator, acoustic thermometry, Boltzmann constant, duct, perturbation, waveguide

## Abstract

We examine the perturbations of the modes of an acoustic thermometer caused by circular ducts used either for gas flow or as acoustic waveguides coupled to remote transducers. We calculate the acoustic admittance of circular ducts using a model based on transmission line theory. The admittance is used to calculate the perturbations to the resonance frequencies and half-widths of the modes of spherical and cylindrical acoustic resonators as functions of the duct’s radius, length, and the locations of the transducers along the duct's length. To verify the model, we measured the complex acoustic admittances of a series of circular tubes as a function of length between 200 Hz and 10 kHz using a three-port acoustic coupler. The absolute magnitude of the specific acoustic admittance is approximately one. For a 1.4 mm inside-diameter, 1.4 m long tube, the root mean square difference between the measured and modeled specific admittances (both real and imaginary parts) over this frequency range was 0.018. We conclude by presenting design considerations for ducts connected to acoustic thermometers.

## 1. Introduction

Acoustic thermometers determine the thermodynamic temperature of dilute, high-purity argon or helium by measuring the acoustic resonance frequencies of a gas-filled cavity [1, 2, 3, 4, 5, 6, 7]. Because these resonances have high quality factors, one can measure the frequencies with relative uncertainties of 10^−6^ or less. Therefore, uncertainty in determining the thermodynamic temperature results from other factors such as impurities in the gas or imperfections of the model for a particular resonator. The model must account for ducts used to flow gas into and out of the resonant cavity as well as crevices at seams joining sections of the cavity’s wall. At high temperatures, the gas under study may be contaminated by outgassing water, hydrogen, etc. Flowing high-purity gas through the resonator *via* ducts (long, thin tubes) effectively mitigates the problem of outgassing [3, 4, 5]. Ducts are also useful as acoustic waveguides to transmit sound between a resonator at high temperature and transducers located near ambient temperature [8, 9]. This practice circumvents the problem of manufacturing efficient, stable electroacoustic transducers and cables that function at high temperatures.

In this paper, we calculate the perturbations of the acoustic resonances of gas-filled cavities caused by circular ducts used either for gas flow or as acoustic waveguides coupled to remote transducers. First, we review the well-established model for the acoustic admittance of circular ducts based on transmission line theory [10, 11, 12, 13]. We calculate the perturbations to the resonance frequencies and half-widths of the modes of spherical and cylindrical acoustic cavities as functions of the duct’s radius, length, and also as a function of the location of the transducers along the duct’s length. To verify the model for a circular duct, we measured the complex acoustic admittances of a series of circular tubes as a function of length between 200 Hz and 10 kHz using a three-port acoustic coupler. Over this frequency range, the root mean square difference between the measured and modeled specific admittances of a 1.4 mm ID, 1.4 m long tube was 0.018. We conclude by discussing design considerations for ducts leading to acoustic thermometers.

## 2. Acoustic Model for Circular Ducts

See Refs. [12] and [13], and references cited therein, for a detailed description of the acoustic model for a duct with a circular cross section. The model is valid for frequencies below the “cutoff frequency” *f*_co_, the frequency below which only plane waves propagate. If the duct’s radius is *r*_d_ and the speed of sound in the gas is *c*, then *f*_co_ ≈ 0.29 *c*/*r*_d_ or about 140 kHz for ambient air in a 1.4 mm ID duct. We adopt the *e^iωt^* time dependence convention, consistent with Refs. [12] and [13]. We restrict the discussion to ducts with length *l*_d_ >> *r*_d_ and thereby neglect the effects of non-planar flow and sharp corners at the ends of the ducts [12]. Finally, we assume that the cross-sectional area of the duct $A_{d}=\pi r_{d}^{2}$ is a small fraction of the resonator’s surface area.

The model’s central result is that the acoustic pressure ${\tilde{p}}_{\pm }$ and volume velocity ${\tilde{U}}_{\pm }$ of damped traveling waves in a duct are plane waves proportional to *e*^±^*^Γz^*^+^*^iωt^*, where the propagation constant *Γ* is given by

$$\Gamma =\frac{i\omega }{c}\sqrt{\frac{1+(\gamma -1)F_{t}}{1-F_{v}}}.$$ (1)

Furthermore, the ratios ${\tilde{p}}_{+}/{\tilde{U}}_{+}$ and ${\tilde{p}}_{–}/{\tilde{U}}_{–}$ define the characteristic impedance *Z*_0_ of the medium in the duct

$$Z_{0}=\frac{\rho c}{A_{d}}\frac{1}{\sqrt{\left(1-F_{v})[1+(\gamma -1)F_{t}\right]}}.$$ (2)

The real part of *Γ* is a measure of the damping of these traveling waves; *Z*_0_ determines the phase between ${\tilde{p}}_{\pm }$ and ${\tilde{U}}_{\pm }$. The quantities *F*_v_ and *F*_t_ are functions of frequency that account for thermoacoustic dissipation near the duct wall. For a duct with radius *r*_d_, the thermal loss function is

$$F_{t}\equiv \frac{2J_{1}(\zeta_{t})}{\zeta_{t}J_{0}(\zeta_{t})},\;\zeta_{t}=(1-i)r_{d}/\delta_{t}$$ (3)

where *J*_0_ and *J*_1_ are Bessel functions and $\delta_{t}=\sqrt{2\lambda_{t}/(\rho C_{P}\omega )}$ is the thermal boundary layer thickness. The viscous loss function *F*_v_ has the same form as Eq. (3) with *δ*_t_ replaced by the viscous boundary layer thickness $\delta_{v}=\sqrt{2\eta /\rho \omega }$ For the gas properties in these expressions, *ρ* is the density, *c* is the speed of sound, *η* is the shear viscosity, *λ_t_* is the thermal conductivity, *C_P_* is the isobaric heat capacity per unit mass, *C_V_* is the isochoric heat capacity per unit mass, and *γ* is the heat capacity ratio *C_P_*/*C_V_*. *F*_v_ and *F*_t_ are complex-valued functions of *δ*_v_/*r*_d_ and *δ*_t_/*r*_d_, respectively. The length scales *δ*_t_ and *δ*_v_ are not independent but are related via the Prandtl number *Pr* = *η C_P_*/*λ*_t_ by the expression $\delta_{v}=\delta_{t}\sqrt{Pr}$. Since *Pr* for monatomic gases is a weak function of temperature and pressure, we describe the damping of waves in a duct in terms of *δ*_t_/*r*_d_ only.

The model for finite-length tubes includes reflections from the ends. The interference between these counter-propagating waves produces standing waves at particular frequencies. Such tube resonances pose a problem if they occur too close in frequency to the cavity’s modes used for thermometry and are insufficiently damped. To model a tube with finite length, we use a lumped-element equivalent circuit defined by a T-network as described in Ref. [12]. The input impedance of a tube with length *l*_d_, terminated by an acoustic impedance *Z*_T_ is

$$Z_{\text{in}}=Z_{0}\frac{Z_{T}+Z_{0}\tanh\left(\Gamma l_{d}\right)}{Z_{0}+Z_{T}\tanh\left(\Gamma l_{d}\right)}.$$ (4)

In the following sections, we investigate the perturbation that such a tube or combinations of tubes has on the modes of acoustic resonators.

## 3. Perturbation of the Modes of an Acoustic Cavity by Ducts

Consider the acoustic modes of a gas in a closed cavity. The acoustic velocity potential Ψ for the mode *N* is a solution to the homogeneous Helmholtz Equation

$$\nabla^{2}\Psi_{N}+\kappa_{N}^{2}\Psi_{N}=0$$ (5)

and satisfies a set of boundary conditions defined by the properties of the gas and the shape of the cavity. [The “acoustic” fields (velocity, pressure, and temperature) are the propagating solutions of the linearized equations of motion. The diffusing solutions (thermal and shear evanescent waves) are important near the boundary and define the acoustic boundary layers.] We assume here that Ψ*_N_* and the eigenvalue *κ_N_* are known (either from analytical or numerical calculation) and describe the mode of our “unperturbed” resonator. For simplicity, we assume that the mode is non-degenerate. Acoustic resonators typically have holes in the wall to admit/remove gas and ports for acoustic transducers. For measurements at extreme temperatures, it may be necessary to use small tubes (ducts) as acoustic waveguides to convey sound to and from remote transducers. Changes to the cavity wall such as these change the boundary conditions on the acoustic wave in the vicinity and, therefore, slightly alter Ψ*_N_* and *κ_N_*. For metrology applications, these perturbations must be quantified with high accuracy.

We focus on spherical, quasi-spherical, and cylindrical cavities. Each shape has advantages. Spherical cavities are characterized by a single length (the radius). Quasi-spherical cavities are nearly spherical cavities with a known shape perturbation that splits the degeneracy of the microwave modes and the non-radial acoustic modes of a perfect sphere. The radially-symmetric modes of a gas-filled quasi-spherical cavity are affected by the shape perturbation only in the second order. Thus, the first-order results in this paper for spherical cavities apply to quasi-spherical cavities as well. Since the wave velocity of a radial mode in a spherical cavity is normal to the cavity’s wall, there is no viscous boundary layer dissipation. Therefore, the quality factors (*Q*) of radial modes in spherical cavities are substantially higher than the *Q*s of modes in non-spherical cavities with the same volume.

The modes of a fixed-length cylindrical cavity are determined by two parameters: its radius and its length. An acoustic interferometer is a cylindrical resonator in which one end is a moveable piston, so that the cavity’s length is variable. The interferometer is operated at a fixed frequency while the length is varied through a succession of resonances [14]. With the interferometer, the speed of sound is determined not from an absolute length but from the measured displacement of the piston between successive resonances. Furthermore, since the measurements are at fixed frequency, the perturbations from the end plates cancel out to first order. An alternative to the interferometer, which avoids the complications of a moveable piston, uses two cylindrical resonators in which the length of one is twice the length of the other. The modes of the shorter cavity occur at the same frequencies as the even-order modes of the longer cavity. The combined measurements from both cavities at the same frequencies also have the advantage that the perturbations from the endplates cancel to first order.

The theory for calculating boundary perturbations has been published elsewhere [15]. From first-order perturbation theory, the shift of the eigenvalue *κ_N_* due to a non-uniform surface admittance is

$$\frac{\Delta K_{N}}{\kappa_{N}}\approx \frac{i\omega }{2c\kappa_{N}^{2}}\frac{\int_{S}\Psi_{N}^{2}y\left(\omega ,r_{s}\right)dS}{\int_{V}\Psi_{N}^{2}\;dV}$$ (6)

where *y*(*ω*, **r***_S_*) is the specific acoustic admittance at point **r***_S_* on the boundary. In terms of the acoustic velocity ${\tilde{u}}_{\mathrm{ac}}$ and the acoustic pressure $\tilde{p}$, *y*(*ω*, **r***_S_*) is defined as

$$y\left(\omega ,r_{S}\right)=\frac{\rho c\hat{n}\cdot {\tilde{u}}_{\text{ac}}\left(\omega ,r_{S}\right)}{\tilde{p}\left(\omega ,r_{S}\right)}$$ (7)

where $\hat{n}$ is the outward pointing normal unit vector at **r***_S_*.

For the ideal resonator, the walls are assumed to have zero admittance. The presence of the duct changes the shape from the perfect resonator and therefore changes the mode wavenumbers, because the acoustic wave propagates into the duct and may be partially reflected back into the resonator. Furthermore, a lossy duct causes additional thermoacoustic dissipation. Thus, the acoustic wave in the resonator locally “feels” a larger admittance at the entrance to the duct than at the cavity’s wall. If the duct’s diameter is small compared to the cavity’s diameter, then the long-wavelength acoustic waves will not vary appreciably over the cross section of the duct; therefore, we can expand Ψ about the duct position **r**d and use the average admittance of the duct over its cross section ${\bar{y}}_{d}$. The leading term in the approximation to the surface integral in Eq. (6) is

$$\int_{S}{\left[\Psi_{N}\left(r_{S}\right)\right]}^{2}y\left(\omega ,r_{S}\right)dS\approx {\bar{y}}_{d}\left(\omega \right){\left[\Psi_{N}\left(r_{d}\right)\right]}^{2}A_{d}.$$ (8)

The wavenumber for the *n*th radial mode of a spherical resonator with radius a is changed by a fractional amount, from Eq. (6),

$$\frac{\Delta K_{n}}{\kappa_{n}}\approx \frac{i{\bar{y}}_{d}A_{d}}{4\pi a^{2}z_{0n}},$$ (9)

where *z*_0_*_n_* denotes the *n*th zero of *dj*_0_(*x*)/*dx*, *n* = 2, 3, 4, …. Because of the spherical symmetry, the perturbation of the radial-mode wavenumbers does not depend on the location of the duct.

For the *l*th longitudinal mode of a cylindrical resonator with radius *R* and length *L*, Eq. (6) becomes

$$\frac{\Delta K_{l}}{\kappa_{l}}\approx \frac{i{\bar{y}}_{d}A_{d}}{l\pi^{2}R^{2}}\cos^{2}\left(l\pi z_{d}/L\right)$$ (10)

where 0 ≤ *z*_d_ ≤ *L* is the axial location of the duct and *l* = 1, 2, 3, …. The first-order perturbation of longitudinal modes when the duct mounted on an endplate (*z*_d_ = 0, *L*) is independent of where on the endplate the duct is located. The position of the duct *z* d must be chosen carefully based on the duct’s purpose. For ducts used with remote transducers, there is a competition between the desire for small perturbations and the desire for efficient coupling with the acoustic modes of the resonator. The perturbation will be smallest when the duct is placed near a pressure node, i.e., 2*lz*_d_/*L* = 1, 3, 5, ….; however, at this location the coupling between the mode and the transducers is least efficient. The perturbation will be largest when the duct is placed near a pressure anti-node, i.e., 2*lz*_d_/*L* = 0, 2, 4, 6,… and the coupling will be most efficient. Node placement is a good choice if the duct’s purpose is to flow gas into and out of the resonator; however, it may be a bad choice if the duct is a waveguide leading to a remote transducer. When the cavity’s length is fixed, the locations of the pressure nodes and anti-nodes on the cylindrical wall depend on the mode. With an interferometer, the cavity length is varied between two extremes *L*_min_ and *L*_max_, and the frequency (and therefore wavelength) is held constant. Therefore, the locations of the pressure nodes and anti-nodes are fixed in space. This feature of the interferometer suggests that an arrangement where the acoustic waveguides are located on the fixed endplate to maximize the coupling to all the modes, and the fill ducts are attached to the side of the resonator at *z*_d_ = *L*_min_/2 at the pressure node. The disadvantage to this arrangement is that it only works for the odd-symmetry modes, which are susceptible to the effects of center-of-mass motion. A better method is to locate the fill ducts and the waveguides on the endplates, study only the even-symmetry modes (to avoid center-of-mass motion), and rely on the cancellation of the first-order perturbations.

In the following sections, we model the acoustic admittance of several duct geometries. Then we estimate the perturbations due to the duct on the radial modes of a 5 cm radius spherical resonator and on the longitudinal modes of a 5 cm radius acoustic interferometer with variable length between 10 cm and 20 cm.

### 3.1 Infinite-Length Duct With Uniform ID

**Fig. 1:**
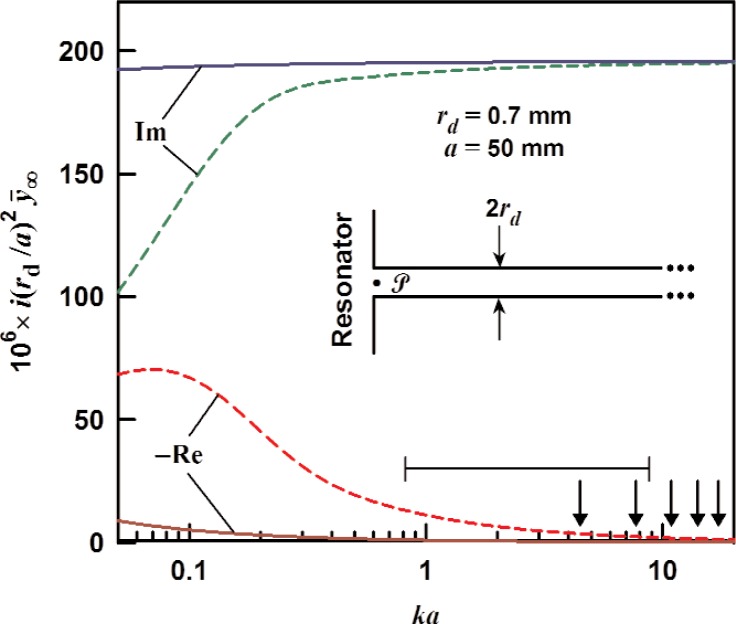
The specific acoustic admittance of an infinite duct, from Eq. (11) multiplied by *i* (*r*_d_/*a*) ^2^, as a function of *ka*. The duct is assumed to be filled with argon at 0.02 MPa (dashed) and 0.4 MPa (solid) at 273 K. − Re: − 1 × real part, Im: imaginary part. The model assumes *r*_d_ = 0.7 mm and the (spherical or cylindrical) resonator radius *a* = 50 mm. The arrows in the lower right corner locate the first five radial modes of a sphere. The horizontal bar shows the range that will excite longitudinal modes of an interferometer with length *L* variable between 9 cm and 19 cm.

We first consider an infinitely long duct with inner radius *r*_d_. An acoustic wave entering the duct (point P in the inset in Fig. 1) travels to the right and damps away with no reflection back to the resonator. As stated in the introduction, the input impedance for such a duct is just the characteristic impedance given in Eq. (2). The specific acoustic admittance for the infinite tube is, therefore,

$${\bar{y}}_{\infty }={\bar{y}}_{0}=\frac{\rho c}{A_{d}}\frac{1}{Z_{0}}=\sqrt{\left[1+\left(\gamma -1\right)F_{t}\right]\left(1-F_{v}\right)}.$$ (11)

The perturbation of a resonator mode, calculated from Eqs. (9) and (10), shifts the mode’s wavenumber (and resonance frequency) by an amount proportional to $\text{Re}\left(i{\bar{y}}_{\infty }r_{d}^{2}/a^{2}\right)$ and increases the half-width by an amount proportional to $\text{Im}\left(i{\bar{y}}_{\infty }r_{d}^{2}/a^{2}\right)$. The wide-tube approximation *r*_d_≫*δ*_t_ to Eq. (11) is

$${\bar{y}}_{\infty }\approx 1-(1-i)\left(1+\sqrt{\mathrm{Pr}}-\gamma \right)\frac{\delta_{t}}{2r_{d}}+O{\left(\frac{\delta_{t}}{r_{d}}\right)}^{2}.$$ (12)

We recommend caution when using Eq. (12) for noble gases because the imaginary part is subject to large errors even when *δ*_t_/*r*_d_ is as small as 0.1. In practice, however, the error in Re(Δ*K*/*κ*) introduced by the wide tube approximation is less than 1 × 10^−6^ when *a*/*δ*_t_ > 200 for the (0,2) mode of a sphere or R/*δ*_t_ > 400 for the first longitudinal mode of a cylinder.

Figure 1 shows the calculated admittance of an infinite duct (*r*_d_ = 0.7 mm) filled with argon at 273 K for pressures of 0.02 MPa (dashed curves) and 0.4 MPa (solid curves). The normalized admittance $i{\left(r_{d}/a\right)}^{2}{\bar{y}}_{\infty }$ is plotted as a function of *ka*. Here, *a* is either the radius of a spherical resonator or the radius of a cylindrical resonator and is assumed to be 50 mm in either case. The arrows in the figure locate radial modes of a sphere, and the horizontal bar shows the range of *ka* that will excite longitudinal modes of an interferometer with length *L* in the range 10 cm < *L* < 20 cm. We estimate $\text{Re}\left(i{\bar{y}}_{\infty }r_{d}^{2}/a^{2}\right)$ for *ka* =1 to be −11.2 × 10^−6^ and −1.0 ×10^−6^ at 0.02 MPa and 0.4 MPa, respectively, whereas $\text{Im}\left(i{\bar{y}}_{\infty }r_{d}^{2}/a^{2}\right)$ is, respectively, 1.91 × 10 and 1.95 × 10^−4^. For the (0,2) mode of a sphere (*ka* ≈ 4.49), we estimate $\text{Re}\left(i{\bar{y}}_{\infty }r_{d}^{2}/a^{2}\right)$ to be −3.6 × 10^−6^ and −0.4 × 10^−6^ at 0.02 MPa and 0.4 MPa, respectively; $\text{Im}\left(i{\bar{y}}_{\infty }r_{d}^{2}/a^{2}\right)$ is 1.94 ×10^−4^ and 1.96 ×10^−4^, respectively.

### 3.2 Two-Stage Infinite Tube

**Fig. 2:**
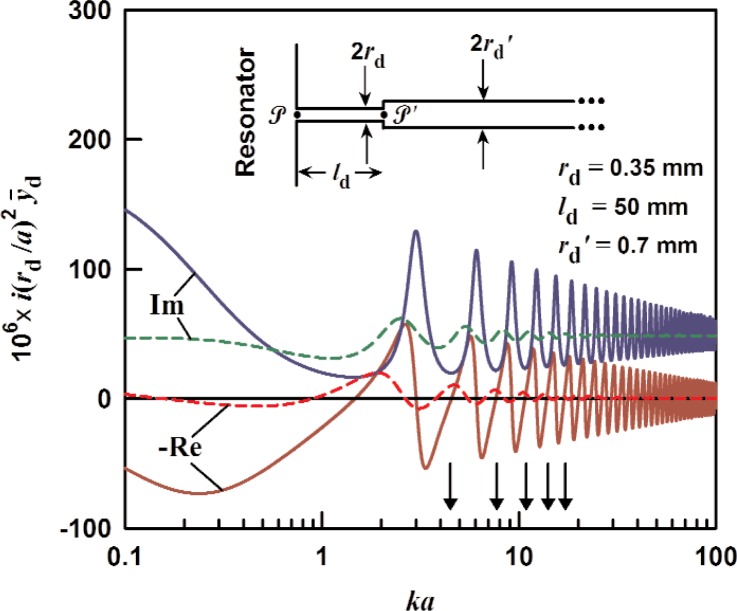
The specific acoustic admittance for the two-stage infinite tube, from Eq. (16) multiplied by *i*(*r*_d_/*a*)^2^, as a function of *ka*. The duct is assumed to be filled with argon at 0.02 MPa (dashed) and 0.4 MPa (solid). The locations of the first 5 radial modes of a sphere are indicated by arrows.

We now examine a two-stage infinite tube shown in the inset in Fig. 2. A smaller diameter duct with radius *r*_d_ and length *l*_d_ is inserted between the resonator and the infinite duct discussed in Sec. 3.1. The wave present in the short duct will be partially reflected at points P and $P^{\prime }$ giving rise to standing waves at specific frequencies. Neglecting the effects on the acoustic field of the abrupt transitions at the duct ends, we assume that the pressure and volume velocity are continuous 1-dimentional functions of *z*. The pressure and volume velocity in the duct are

$$p_{d}=p_{L}e^{\Gamma z}+p_{R}e^{-\Gamma z},\;U_{d}=-\frac{1}{Z_{0}}\left[p_{L}e^{\Gamma z}-p_{R}e^{-\Gamma z}\right]$$ (13)

respectively, and in the infinite tube we have

$$p_{d}{}^{\prime }=p_{R}{}^{\prime }e^{-\Gamma^{\prime }\left(z-l_{d}\right)},\;U_{d}{}^{\prime }=\frac{p_{R}{}^{\prime }e^{-\Gamma^{\prime }\left(z-l_{d}\right)}}{Z_{0}{}^{\prime }}.$$ (14)

We match the impedances in the two ducts at point $P^{\prime }$, i.e., ${\left(U_{d}/p_{d}\right)}_{z}{}_{=l_{d}}={\left(U_{d}{}^{\prime }/p_{d}{}^{\prime }\right)}_{z}{}_{=l_{d}}$and then use the impedance at point P, *Z*_d_ = (*U*_d_/*p*_d_)*_z_*_= 0_, to eliminate *p*_R_/*p*_L_. Rearranging, we obtain

$$Z_{d}=Z_{0}\frac{Z_{0}{}^{\prime }+Z_{0}\tanh\left(\Gamma l_{d}\right)}{Z_{0}+Z_{0}{}^{\prime }\tanh\left(\Gamma l_{d}\right)}.$$ (15)

Equation (15) has the same form as the equation for a finite-length lossy transmission line (i.e., the small duct) terminated by the impedance *Z*_0_′. Derivation of this expression from an equivalent T-network is given in the next section. The specific acoustic admittance at the duct entrance is

$${\bar{y}}_{d}={\bar{y}}_{0}\frac{Z_{0}+Z_{0}{}^{\prime }\tanh\left(\Gamma l_{d}\right)}{Z_{0}{}^{\prime }+Z_{0}\tanh\left(\Gamma l_{d}\right)}.$$ (16)

with the definition ${\bar{y}}_{0}\equiv \rho c/\left(A_{d}Z_{0}\right)$. Figure 2 shows the specific admittance for the two-stage infinite tube (*r*_d_ = 0.35 mm, *l*_d_ = 50 mm) from Eq. (16) scaled by *i*(*r*_d_/*a*)^2^, where *a* = 50 mm is either the radius of a spherical cavity or the radius of a cylindrical cavity. The acoustic medium is assumed to be argon at 273 K and 0.02 MPa (dashed) and 0.4 MPa (solid). For comparison, the specific admittance of the uniform infinite tube from Eq. (11), scaled by $i{\left(r_{d}{}^{\prime }/a\right)}^{2}$, where $r_{d}{}^{\prime }$ = 0.7 mm, is also shown. The peaks in ${\bar{y}}_{d}$ indicate damped resonances. The left-most peak, near *ka* = 0, is a Helmholtz mode in which gas moves asymmetrically between the resonator and the infinite tube through the small duct. The other peaks are resonances in the small duct. Between the resonances are regions where the admittance is small.

The length of the short tube in Fig. 2 was chosen such that the modes of the spherical cavity fall in between the modes of the duct where the admittance is near zero. By comparison, the characteristic impedances of the long tube and of the cavity are small compared to the characteristic impedance of the short duct, so the duct itself is nearly a half-wave resonator with resonances when *ka* ≈ *mπa*/*l*_d_, with *m* a positive integer. The anti-resonances (minimum amplitude) occur when *ka* ≈ (*m+*½)*πa*/*l*_d_. The low-lying radial modes of a spherical cavity will be close to these anti-resonances if *l*_d_ = *a*.

For a cylindrical cavity, the optimum arrangement is different. If $r_{d}{}^{\prime }>r_{d}$, as in the inset of Fig. 2, the odd-symmetry longitudinal modes can be placed near the anti-resonances if, for example, *l*_d_ = 1.5 *L*. However, both the even and odd-symmetry modes cannot be placed near anti-resonances. On the other hand, if we choose $r_{d}{}^{\prime }<r_{d}$, then the short duct will be nearly a quarter-wave resonator with anti-resonances when *k_m_* = *mπ*/*l*_d_. If we further choose *l*_d_ = 1.5 *L*, the even-symmetry modes of the cavity fall between the modes of the short duct.

### 3.3 Finite-Length Tube With Uniform ID

**Fig. 3:**
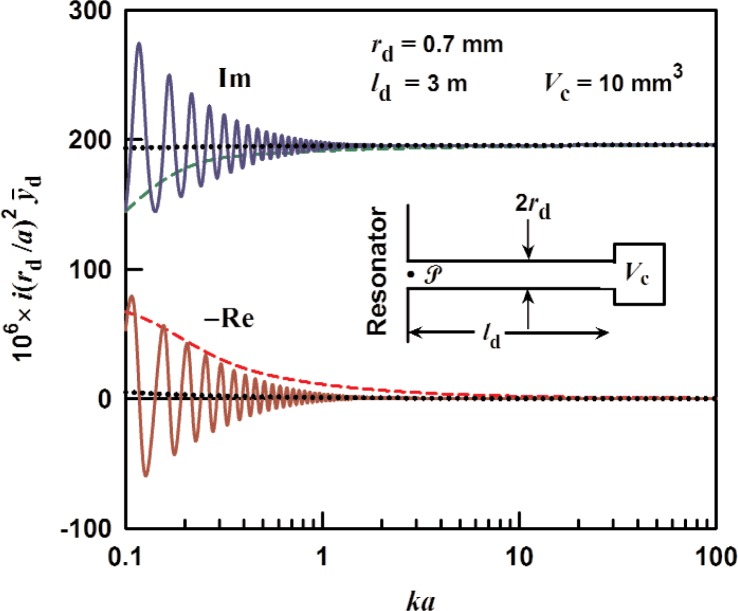
The specific acoustic admittance for a terminated tube, from Eq. (18) multiplied by *i*(*r*_d_/*a*)^2^, as a function of *ka*. The duct is assumed to be filled with argon at 0.02 MPa (dashed), 0.4 MPa (solid) versus *ka*. The admittance for an infinite tube is also shown for 0.4 MPa (dotted) for comparison.

The inset in Fig. 3 shows a sketch of a tube (with diameter 2*r*_d_ and length *l*_d_) terminated by an impedance *Z*_T_. This arrangement is suitable for a fill duct in which the terminal impedance results from a chamber with volume *V_c_*, such as a valve. In this case, since the chamber’s dimensions are smaller than an acoustic wavelength, *Z*_T_ will be the impedance of the chamber volume, given by

$$Z_{T}=\frac{\rho c^{2}}{i\omega V_{c}\left[1+\frac{1}{2}\left(1-i\right)\left(\gamma -1\right)S_{c}\delta_{t}/V_{c}\right]},$$ (17)

where *S_c_* is the chamber’s surface area. If the duct is open into a pressure vessel, then the termination impedance will likely be dominated by the radiation impedance except perhaps near the resonances of the pressure vessel. Radiation impedance is discussed in Sec. 4. The specific acoustic admittance for this arrangement is

$${\bar{y}}_{d}={\bar{y}}_{0}\frac{Z_{0}+Z_{T}\tanh\left(\Gamma l_{d}\right)}{Z_{T}+Z_{0}\tanh\left(\Gamma l_{d}\right)}.$$ (18)

The normalized specific admittance $i{\bar{y}}_{d}r_{d}^{2}/a^{2}$ is plotted in Fig. 3 as a function of *ka* for a tube 3 meters long. The acoustic medium is assumed to be argon at 0.02 MPa and 0.4 MPa. The oscillations in the admittance in Fig. 3 are due to resonances that occur in the tube. The locations of the resonances are sensitive to the termination impedance, however the envelope defined by the peak-to-peak oscillations is determined primarily by the damping in the tube. Therefore, this envelope gives an estimate of the uncertainty in the admittance if the termination volume *V_c_* is unknown. The uncertainty in the admittance defined by the envelope is approximately

$$\sigma_{\bar{y}}=\pm 2{\left(\frac{r_{d}}{a}\right)}^{2}e^{-2l_{d}/l_{a}},$$ (19)

where the attenuation length *l*_a_ ≡ 1/Re(Γ) is the characteristic distance that the wave travels in the tube before it damps out (see Sec. 3.5). The attenuation length is about 0.6 m for sound at the frequency of the lowest radial mode (*ka* = 4.49) in a tube with an inner radius of 0.7 mm filled with argon at 273.16 K and 0.4 MPa pressure. For the dimensions used in Fig. 3 and 0.4 MPa, we estimate $\sigma_{\bar{y}}\approx \pm 5\times 10^{-6}$and $\sigma_{\bar{y}}\approx \pm 1\times 10^{-8}$ at *ka* = 0.8 and 4.5, respectively.

A special case of an open-ended tuned-length tube is worth considering here. Resonators are usually placed in a pressure vessel to reduce the dimensional changes caused by changes of pressure and to provide thermal isolation. A short open tube can provide a means of changing the gas pressure in the resonator quickly, instead of the much slower method through a long tube. We caution, though, that a duct open to a pressure vessel may cause difficulties if an acoustic mode of the vessel overlaps with a mode of either a spherical or cylindrical cavity.

### 3.4 Two-Stage Tube With Termination

**Fig. 4:**
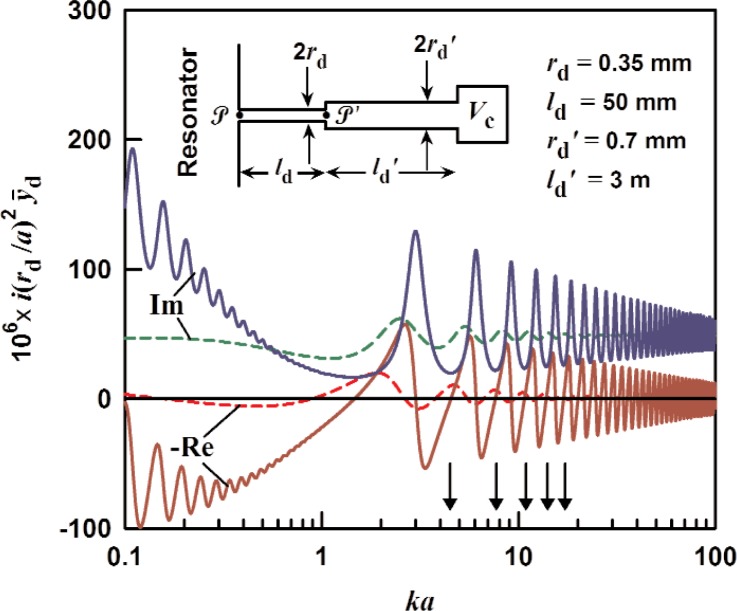
The specific acoustic admittance of a terminated 2-stage tube, from Eq. (20) multiplied by *i*(*r*_d_/*a*)^2^, as a function of *ka*. The duct is assumed to be filled with argon at 0.02 MPa (dashed), 0.4 MPa (solid) versus *ka*.

We now investigate a 2-stage tube in which the opening into the resonator is through a short duct with a smaller diameter, as shown in the inset in Fig. 4. We can use the methods in the previous sections to write the admittance at point P as

$${\bar{y}}_{d}={\bar{y}}_{0}\frac{{\left(r_{d}{}^{\prime }/r_{d}\right)}^{2}{\bar{y}}_{d}{}^{\prime }+{\bar{y}}_{0}\tanh\left(\Gamma l_{d}\right)}{{\bar{y}}_{0}+{\left(r_{d}{}^{\prime }/r_{d}\right)}^{2}{\bar{y}}_{d}{}^{\prime }\tanh\left(\Gamma l_{d}\right)}$$ (20)

where ${\bar{y}}_{d}{}^{\prime }$ is the admittance at point $P^{\prime }$ from Eq. (18). In Eq. (20), ${\bar{y}}_{0}$ and the unprimed quantities with subscript d refer to the short duct. The results are shown in Fig. 4.

### 3.5 Design Suitable for Remote Transducers

When tubes are used as acoustic waveguides to transmit sound between the resonator and remote transducers, the perturbation due to the waveguides must be weighed against the signal-to-noise ratio. If not properly addressed, these issues may result in an unacceptably large uncertainty with which the speed of sound or temperature is measured. For example, if a detector microphone is placed at the end of the waveguide, as in the inset of Fig. 3, resonances will occur in the waveguide at specific frequencies due to the impedance mismatch between the duct and the transducer. Weakly-damped resonances in the waveguides may couple to the modes of the resonator causing significant frequency shifts; furthermore, the unwanted resonance response of the tube that may be difficult to deconvolve from the resonator’s response. The reflections and the tube resonances can be strongly damped if the waveguide is sufficiently long, but then the attenuation of the desired signal will reduce the signal-to-noise ratio. These and other issues will be discussed in more detail in a forthcoming publication [16]. We give the basic concepts below.

**Fig. 5:**
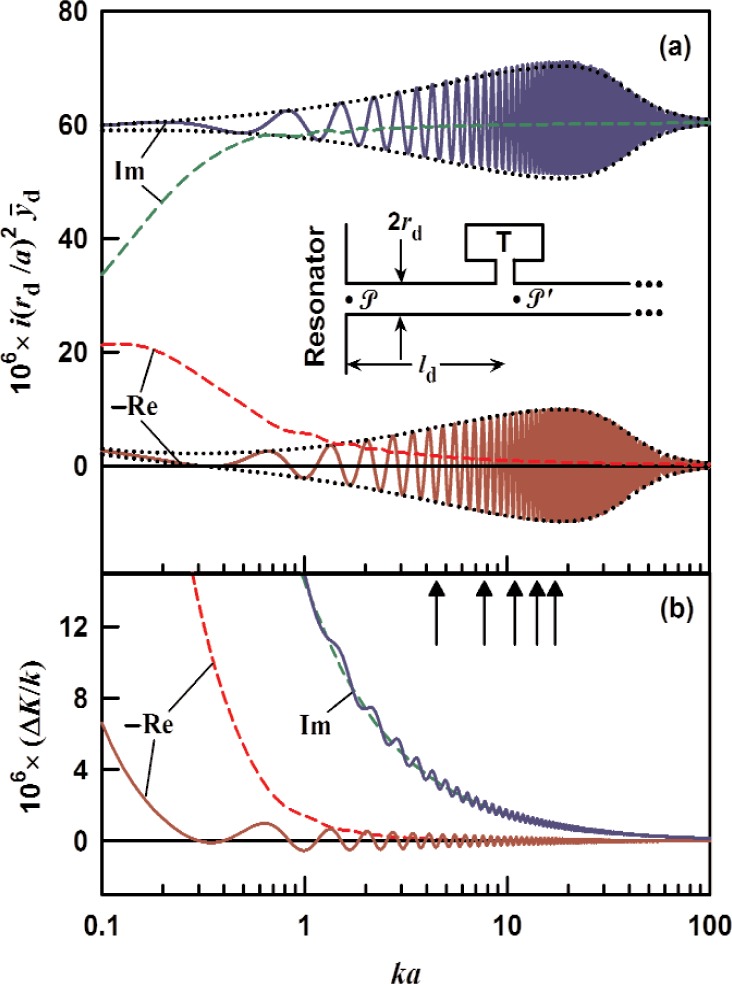
An infinitely long waveguide (*r*_d_ = 0.7 mm) with a transducer (T) located *l*_d_ = 0.4 m from the resonator is shown in the inset. The transducer’s impedance is described in the text. Results for argon at 273 K and pressures of 0.02 MPa (dashed) and 0.4 MPa (solid) in a 9 cm resonator are shown [9]. (a) The real part (red, dark red) and the imaginary part (green, blue) of the normalized specific admittance from Eq. (21). (b) The real part (red, dark red) and the imaginary part (green, blue) of the fractional perturbation from Eq. (9).

A successful design, implemented by Ripple, et al. [9] for the detector and its waveguide, is to mount the detector (T) as a branch with impedance *Z*D on a long waveguide at an intermediate distance *l*_1_ from the resonator, as shown in the inset of Fig. 5a. For this split-tube design, the admittance at P is

$${\bar{y}}_{d}={\bar{y}}_{0}\frac{y_{D}+{\bar{y}}_{d}{}^{\prime }+{\bar{y}}_{0}\tanh\left(\Gamma l_{d}\right)}{{\bar{y}}_{0}+\left(y_{D}+{\bar{y}}_{d}{}^{\prime }\right)\tanh\left(\Gamma l_{d}\right)}$$ (21)

where $y_{D}\equiv \left(pc/A_{d}\right)Z_{D}^{–1}$, and ${\bar{y}}_{d}{}^{\prime }$ is the admittance at $P^{\prime }$ given by

$${\bar{y}}_{d}{}^{\prime }={\bar{y}}_{0}\frac{1+{\bar{y}}_{0}\left(A_{d}Z_{T}/\rho c\right)\tanh\left(\Gamma l_{d}{}^{\prime }\right)}{{\bar{y}}_{0}\left(A_{d}Z_{T}/\rho c\right)+\tanh\left(\Gamma l_{d}{}^{\prime }\right)}.$$ (22)

If *l*_d_′ is sufficiently large, then a wave at $P^{\prime }$ traveling to the right will be damped out before it reaches the end of the waveguide. The characteristic length for damping is the attenuation length *l*_a_, the distance a wave must travel for the acoustic pressure to be attenuated to 1/*e* of its initial value. Thus, if $l_{d}{}^{\prime }\gg l_{a}$ then ${\bar{y}}_{d}{}^{\prime }\approx {\bar{y}}_{0}$ from Eq. (22), and ${\bar{y}}_{d}$ in Eq. (21) will be insensitive to *Z*_T_.

The scaled admittance $i{\left(r_{d}/a\right)}^{2}{\bar{y}}_{d}$ in the limit $l_{d}{}^{\prime }\to \infty$ is plotted in Fig. 5a for Ripple’s sphere, which had a radius of 9 cm. The transducer Ripple used was a commercially-available micromachined microphone with an estimated internal volume of 8.8 mm^3^ and internal surface area of 60 mm^3^. The transducer was connected to the waveguide through a short duct with radius *r*_n_ = 0.5 mm and length *l*_n_ = 0.6 mm. We approximated the transducer impedance *Z*D by the sum of the short duct’s impedance $Z_{\text{neck}}\approx \left(pc/\pi \;r_{n}^{2}\right)ikl_{n}/\left(1–F_{\text{vn}}\right)$, where *F*_vn_ is the viscous loss function, and the volume’s impedance *Z*_vol_ from Eq. (17) with *V*_c_ = 8.8 mm^3^ and surface area *S*_c_ = 60 mm^2^. The oscillations of the admittance in Fig. 5a indicate resonances in the short section (*l*_d_ = 0.4 m, *r*_d_ = 0.7 mm) between the resonator and the transducer. The details of these oscillations are controlled by the reflection coefficient at point $P^{\prime }$, $ℛ=-y_{D}\left(y_{D}+2{\bar{y}}_{0}\right)$. The envelope for these oscillations (also shown in Fig. 5a) has the form

$$i{\left(r_{d}/a\right)}^{2}{\bar{y}}_{d}≃i{\left(r_{d}/a\right)}^{2}{\bar{y}}_{0}\pm 1\left|ℛ\right|e^{-2l_{d}/l_{a}}\left(1+i\right)i{\left(r_{d}/a\right)}^{2}{\bar{y}}_{0}.$$ (23)

The shape of the envelope is determined from two competing phenomena: 1) the magnitude of the reflection coefficient |ℛ| increases as *ka* (or the frequency) increases, and 2) the attenuation length decreases as *ka* increases. The attenuation length for a circular duct is

la≡1Re(Γd)≈rd2ka(Pr+γ−1)(caDt)1/2, (24)

where the approximation is valid for *δ*_t_ ≪*r*_d_. Equation (24) tells us that acoustic waves in a duct travel the furthest at low frequencies and high gas densities, all else being equal. The perturbation from the waveguide and the transducer on the acoustic modes is shown in Fig. 5b. We estimate the perturbation of the (0,2) mode’s resonance frequency Re(Δ*K*/*k*) to be ±0.4 × 10^−6^ based on the envelope in Eq. (23).

If both the source and detector are mounted remotely, then the attenuation in both waveguides must be taken into account. An acoustic wave with pressure amplitude ${\tilde{p}}_{S}$ at the source is attenuated as it propagates a distance *l*_S_ down the waveguide to the resonator, gets amplified by the resonance quality factor *Q*_N_, and is again attenuated as it further propagates a distance *l*_D_ down a similar waveguide to the detector where the acoustic pressure is ${\tilde{p}}_{D}$. The ratio of pressures ${\tilde{p}}_{D}/{\tilde{p}}_{S}$ is proportional to $Q_{N}\;e^{–(l_{s}+l_{D})/l_{a}}$, which is useful to estimate the source strength needed to achieve a desired signal-noise ratio.

## 4. Experimental Details

**Fig. 6:**
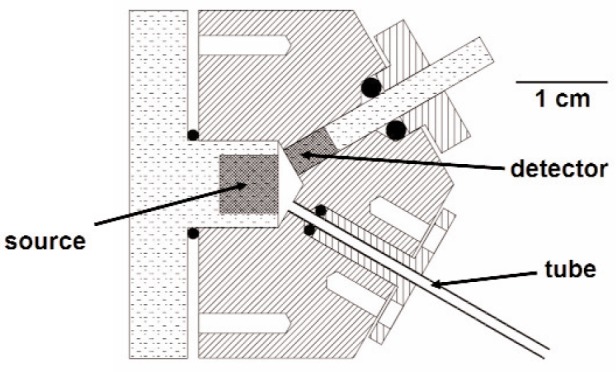
3-port acoustic coupler used to measure the impedance of small tubes.

We studied the acoustic propagation between 200 Hz and 10 kHz in 1.4 mm ID tubes using a 3-port acoustic coupler as shown in Fig. 6. The internal chamber of the coupler was conical with a volume of 134 mm^3^ (determined from dimensional measurements). The sound source was a 6 mm diameter condenser microphone cartridge (Brüel & Kjær^2^ 4135) mounted in a flange and placed in one of the ports. The detector was a 3 mm diameter condenser microphone (Brüel & Kjær 4138) and preamp (Brüel & Kjær 2669) placed in a second port. The third port held the tube under study. The microphones and the tube were positioned flush with the interior surface of the coupler and were secured with elastomer o-rings and a small amount of grease to fill the crevices. The coupler was operated in ambient air. The temperature of the coupler and the air pressure were continuously recorded. The properties of air from NIST’s properties database REFPROP [17] were used to correct for temperature and pressure changes. The admittances of the tubes are determined with a ratio-metric method (described below); therefore, most of the systematic errors due to the properties of air drop out to first order. The tube admittance is a function of *kl*_d_ = 2*πfl*_d_/*c*. Since the frequency is known with high accuracy, the uncertainty of the admittance is dominated by the uncertainty in the quantity *l*_d_/*c*. The uncertainty in the length measurement *u*(*l*_d_) is 0.5 mm, and the relative standard uncertainty of the speed of sound *u*(*c*)/*c* is 0.001.

**Fig. 7:**
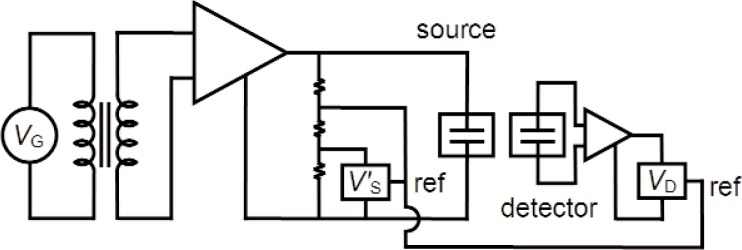
Electronic circuit.

The experimental electronics were arranged as shown in Fig. 7. A sinusoidal drive voltage *V*_G_ at frequency *f*_G_ from a Hewlett-Packard 3225B function generator was amplified and applied to the source microphone. The applied root mean square (rms) voltage was nominally 20 V, but depended on the frequency. The actual rms drive voltage *V*_S_ (scaled by a factor of 0.008 with a voltage divider) was measured at each frequency with a digital lock-in amplifier (Stanford Research SR8350). The signal from the detector microphone *V*_D_ (at frequency 2*f*_G_) was measured with a second lock-in amplifier. Both the detector and source lock-in amplifiers were referenced to the applied drive voltage to eliminate phase shifts from the external electronics. The detector voltage *V*_D_ was proportional to $V_{S}^{2}$, as explained in Appendix A. To remove the frequency dependence of *V*_S_, we normalized the detector voltage by (20 V/*V*_S_)^2^. The acoustic pressure was determined from the expression

$$p_{a}=\left(0.001\;\mathrm{Pa}/\mu V\right)\left(V_{D}\right){\left(20\;V/V_{S}\right)}^{2}.$$ (25)

The observed root mean squared noise in the acoustic pressure was about *u*(*p*_a_) = 0.001 *p*_a_. This noise level is consistent with fluctuations in ambient temperature and pressure on the time scale of the measurement at each frequency, since the temperature and pressure of the air in the coupler and the tubes was not controlled.

We studied 9 different lengths all cut from the same tube: 1601.12 mm, 1400.94 mm, 1200.77 mm, 1000.59 mm, 800.01 mm, 599.84 mm, 399.86 mm, 199.48 mm, and 98.51 mm. (These lengths resulted from fits to the admittance data; however, they all were within the 0.5 mm uncertainty of direct measurements of the length using a steel scale.) We determined the inside diameter of a 3 meter length of the tube from measurements of the pressure drop across the tube produced by a known flow rate of nitrogen gas. The rate of flow through the tube was measured with a mass flow transfer standard (DH Instruments, Molbloc-L, calibrated by the NIST Fluid Metrology Group) attached to the upstream end of the tube. The downstream end of the tube was open to the room. The Molbloc’s downstream pressure transducer was fitted with a two-way valve that enabled us to measure sequentially the pressure at the junction between the Molbloc and the tube, then ambient room pressure. The difference between the two pressures was a measure of the pressure drop across the long tube. We used three flow rates (nominally 74 µmol/s, 220 µmol/s, and 450 µmol/s) in order to check for nonlinear effects. We analyzed the data with the detailed model for flow in a circular tube described by Berg [18]. A small correction due to the increase in kinetic energy at the tube entrance was applied to the flow rate in accordance with Eq. 40 in Ref. [18]. This correction increased the flow rate by at most 1%. Based on these measurements, we determined the tube’s inner diameter to be (1.377 ± 0.005) mm.

The longest tube (1601.12 mm) was used to determine the acoustic source strength *U*_0_ based on the measured acoustic pressure and the calculated impedances. The source strength was then used to determine the tube impedance from the measured acoustic pressure for the other tube lengths.

**Fig. 8:**
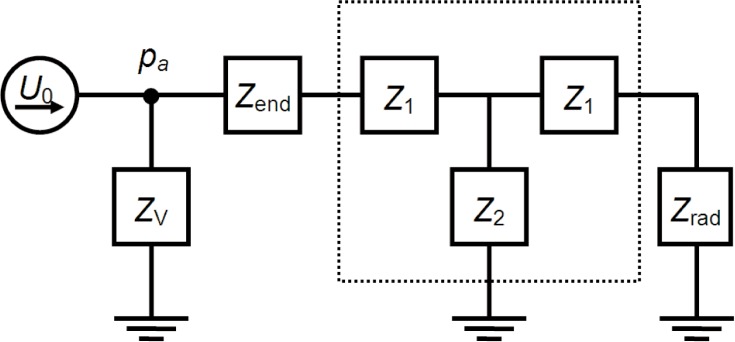
Acoustic model of the acoustic coupler, source, and tube.

The lumped-element model for the acoustic coupler, the source, and the tube is shown in Fig. 8. The acoustic source is modeled as a current source that generates a volume velocity *U*_0_ *e^iωt^*. The acoustic impedance of the coupler volume is denoted *Z*_V_, and the tube impedance is modeled as a waveguide T-network. The small impedances *Z*_end_ and *Z*_rad_ are corrections that account for the imperfect flow fields near the tube end inside the coupler and near the open end, respectively. The detector microphone measures the acoustic pressure *p*_a_ in the coupler volume as indicated in the figure. If we neglect the small end correction *Z*_end_ in Fig. 8, the acoustic pressure in the coupler generated by the source is given by *p*_a_ = *U*_0_*Z*_total_, where the total acoustic impedance seen by the source is

$$\frac{1}{Z_{\text{total}}}=\frac{1}{Z_{V}}+\frac{1}{Z_{\text{tube}}}.$$ (26)

In order to include the end effect impedance *Z*end in Eq. (26), we simply replace *Z*_tube_ with *Z*_tube_ + *Z*_end_.

In the frequency range studied here, the wavelength of sound was always much larger than the coupler’s internal dimensions. Under these conditions, the acoustic impedance of a chamber has been shown to be insensitive to the geometry but dependent simply on the volume and surface area. Therefore, the impedance of the coupler volume was assumed to be given by [19]

$$Z_{V}=\frac{\rho c^{2}}{i\omega V_{c}}\frac{1}{\left[1+\left(1-i\right)\left(\gamma -1\right)\delta_{t}S_{c}/\left(2V_{c}\right)\right]}.$$ (27)

The input impedance of a tube with radius *r*_d_ and length *l*_d_ is given by [see Eq. (4)]

$$Z_{\text{tube}}=Z_{0}\frac{Z_{\text{rad}}+Z_{0}\tanh\left(\Gamma l_{d}\right)}{Z_{0}+Z_{\text{rad}}\tanh\left(\Gamma l_{d}\right)}$$ (28)

where the characteristic impedance *Z*_0_ and the propagation parameter *Γ* include the effects of the thermoacoustic boundary layer in the tube. The radiation impedance *Z*_rad_ has the form (for *kr*_d_ ≪ 1)

$$Z_{\text{rad}}=\frac{\rho \omega }{A_{d}}\left(\delta_{R}+i\delta_{I}\right)$$ (29)

where *δ*_R_ is the orifice resistance parameter and *δ*_I_ is the inertial end correction for sound radiating from the end of an unbaffled open tube. We used the results of Levine and Schwinger [20] for an infinitely thin wall tube

$$\begin{array}{l} \frac{\delta_{I}}{r_{d}}=\varepsilon_{i0}+\varepsilon_{i2}{\left(kr_{d}\right)}^{2},\\ \frac{\delta_{R}}{r_{d}}\approx \frac{1}{4}kr_{d}-\frac{1}{12}\left[\frac{19}{12}-\gamma_{e}-\ln\left(kr_{d}\right)\right]{\left(kr_{d}\right)}^{3} \end{array}$$ (30)

where *ε_i_*_0_ = 0.6127, *ε_i_*_2_ = − 0.1750, and *γ*_e_ is Euler’s constant. For convenience, we define the specific acoustic impedance *z*_rad_ ≡ (*A*_d_/*pc*)*Z*_rad_, and the admittances *y_V_* ≡ (*pc*/*A*_d_)(1/*Z_V_*), and *y*_tube_ = (*pc*/*A*_d_)(1/*Z*_tube_), and *y*_0_ = (*pc*/*A*_d_)(1/*Z*_0_). With these definitions and substitution from Eq. (28), the admittance of the tube is

$$y_{\text{tube}}=y_{0}\frac{1+z_{\text{rad}}y_{0}\tanh\left(\Gamma l_{d}\right)}{z_{\text{rad}}y_{0}+\tanh\left(\Gamma l_{d}\right)}.$$ (31)

**Fig. 9:**
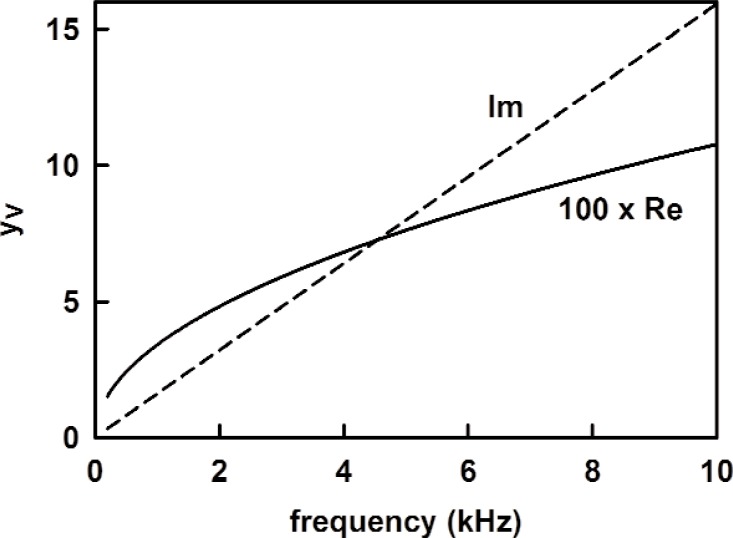
The admittance of the coupler volume *y_V_* = (*ρc* / *A*_d_) (1/*Z_V_*) from Eq. (27) as a function of frequency.

A plot of *y_V_* is shown in Fig. 9.

We attempted to determine the source strength with the tube port plugged, however we were not able to obtain a consistent and repeatable seal around the plug and tubes. Instead, we started with the longest tube (1.6 m) mounted flush with the inner coupler wall, measured the acoustic pressure as a function of frequency, and then cut portions off the tube’s free end to change the length without disconnecting the tube from the coupler. In this way, the baseline conditions at the coupler end were undisturbed and could be reliably eliminated.

**Fig. 10:**
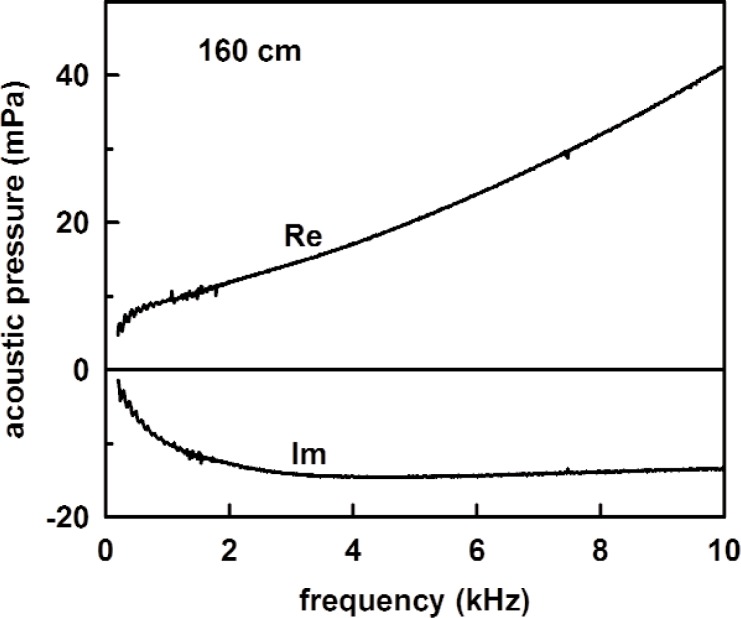
The measured acoustic pressure for the 1.6 m tube.

**Fig. 11:**
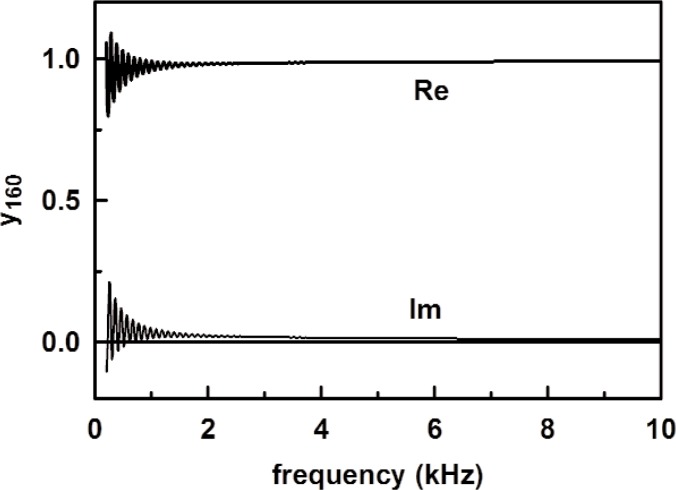
The specific acoustic admittance *y*_160_ for the 1.6 m tube from Eq. (31) as a function of frequency.

**Fig. 12:**
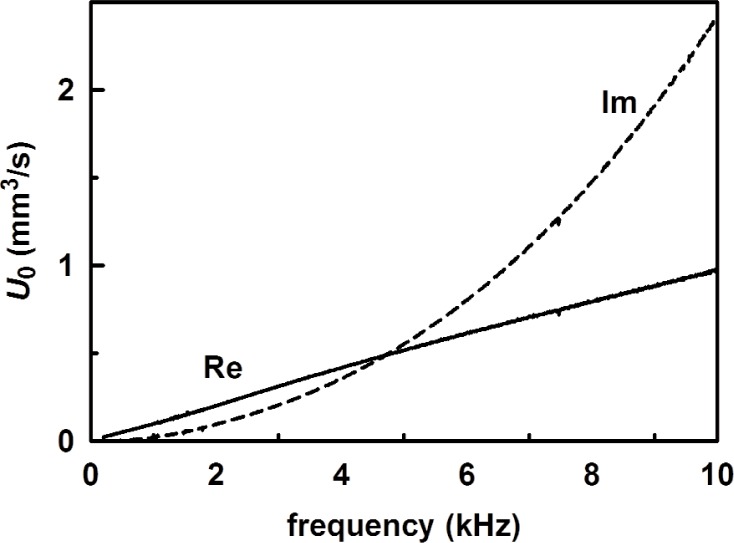
The measured source strength *U*_0_ as a function of frequency.

Figure 10 shows the measured acoustic pressure with the 1.6 m long tube in place. The source strength *U*_0_ was determined from the measured acoustic pressure combined with the calculated impedances of the coupler and tube from Eqs. (26)–(28). In terms of admittances, the source strength is

$$U_{0}=\frac{A_{d}}{\rho c}p_{160}\left(y_{v}+y_{160}\right)$$ (32)

where *p*_160_ is the measured acoustic pressure, *y*_160_ is given by Eq. (31) for the 1.6 m tube. The theoretical tube impedance is plotted in Fig. 11, and the source strength deduced using Eq. (32) from the measured acoustic pressure with no adjustable parameters is shown in Fig 12.

The simple model for the source given in Appendix A predicts a linear frequency dependence for the source strength with a slope that is consistent with the slope of Re(*U*_0_) in Fig. 12. At 1000 Hz, the measured source strength was 0.098 mm^3^/s compared to the predicted value 0.091 mm^3^/s with no adjustable parameters. The simple model in Appendix A does not predict the observed Im (*U*_0_), however. Measurements of the acoustic pressure above 10 kHz revealed that the increase in Re(*p*) with frequency [and therefore the increase in Im(*U*_0_)] is due to the tail of a large resonance at 40 kHz, presumably of the membrane. For all the measurements presented here, the source strength was limited by the microphone’s effective volume and membrane tension, but not limited by the impedance of the coupler or the attached tubes. We therefore used the same source strength function for all the tubes in order to deduce the tube impedances.

After shortening the duct by cutting off a section, leaving the attachment to the coupler unaltered, the acoustic pressure as function of frequency was again measured. The admittance of the tube with new length *l* was determined, with no adjustable parameters, from

$$y_{l}=\left(\frac{\rho c}{A_{d}}\right)\frac{U_{0}}{p_{l}}-y_{V}\;.$$ (33)

**Fig. 13:**
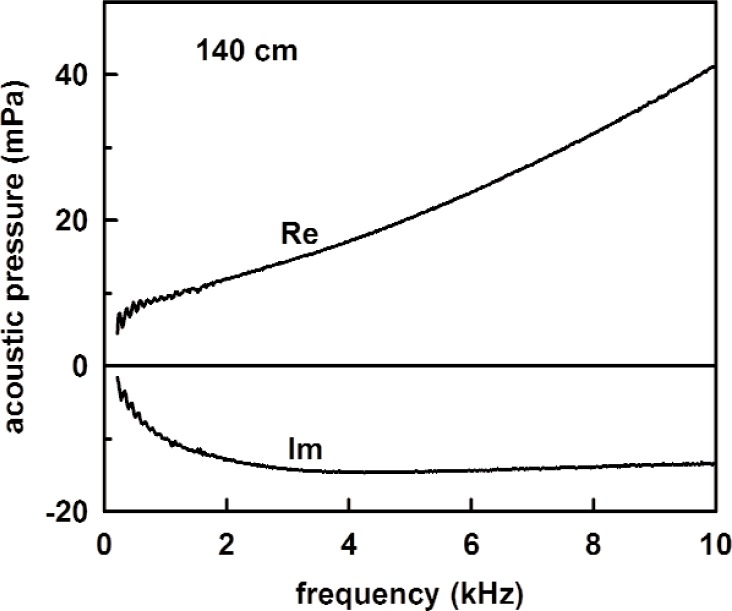
The measured acoustic pressure for the 1.4 m tube.

**Fig. 14:**
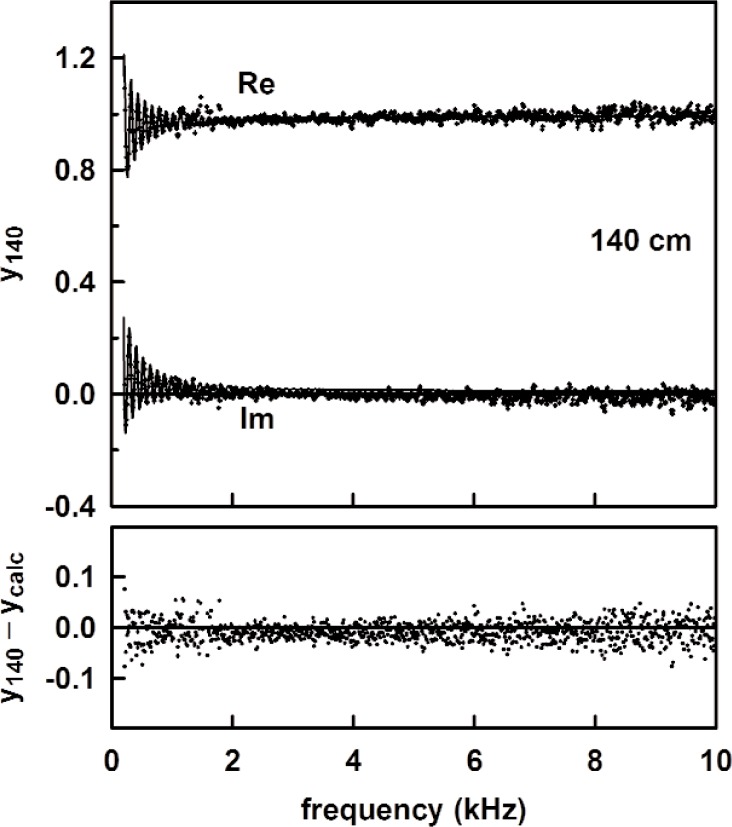
(upper) The measured and calculated admittance for the 1.4 m long tube as functions of frequency. (lower) The difference between the measured and calculated admittance. The standard deviation of the difference is 0.018.

**Fig. 15:**
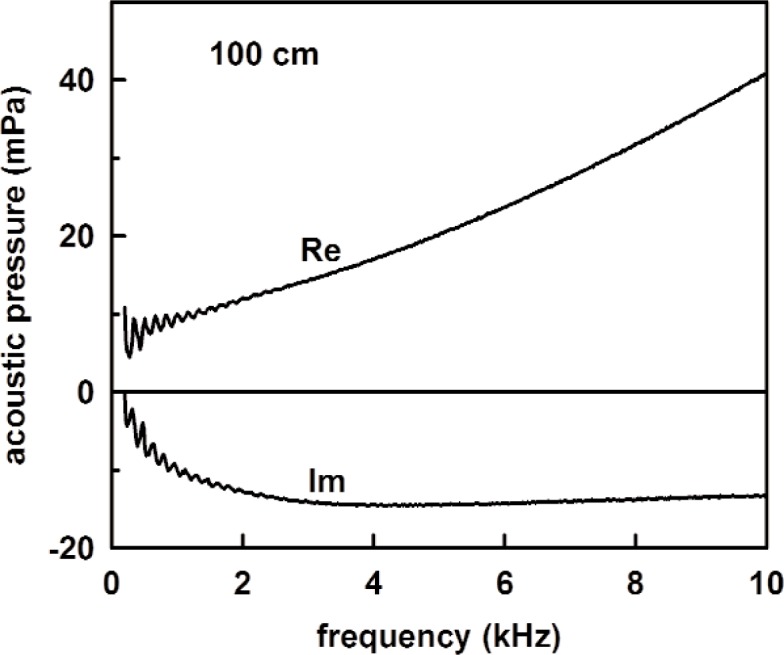
The measured acoustic pressure for 1.0 m tube versus frequency.

**Fig. 16:**
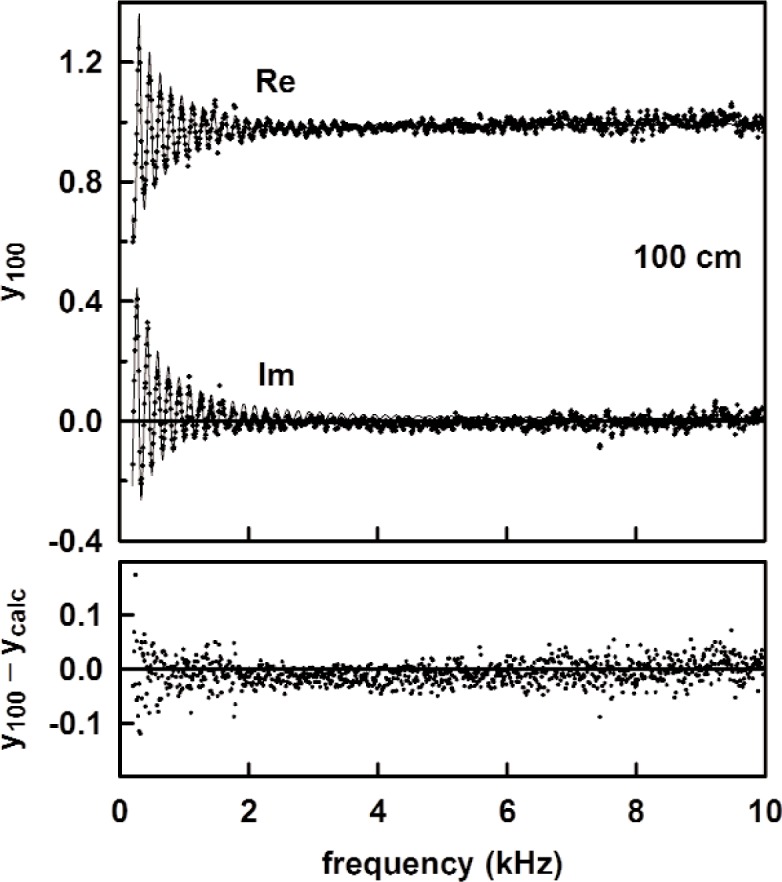
(upper) The measured and calculated admittance for the 1.0 m long tube as functions of frequency. (lower) The difference between the measured and calculated admittance.

Figure 13 shows the measured acoustic pressure for the 1.4 m length, and the measured tube admittance is plotted in Fig. 14 as a function of frequency. Also plotted in Fig. 14 are the theoretical admittance (solid curve) and the deviations between the measured and calculated values. The root mean square difference between the measured and modeled specific admittances (both real and imaginary parts) over this frequency range was 0.018. The observed noise in the measured acoustic pressure mentioned previously accounts for the scatter in the deviation in Fig. 14. Figures 15 and 16 show similar plots of the acoustic pressure and the measured admittance for the 100 cm long tube.

We observed the largest systematic deviations between the measured and predicted admittances of the shortest tubes, where the admittance is more sensitive to errors in the length and to the details of the termination impedance. The attenuation length ***ℓ***_a_ = 1/Re(Γ)is a measure of how far the wave propagates down a long tube before damping out. Lower frequency waves travel further than higher frequency waves: ***ℓ***_a_ = 1.4 m at 200 Hz, whereas ***ℓ***_a_ = 0.22 m at 10 kHz. At 200 Hz, the acoustic pressure at the open end of the 20 cm long tube is attenuated by only 13 % of the acoustic pressure in the coupler. Once the wave leaves the open end, the attenuation length is significantly longer.

Our impedance measurements are comparable to the 1982 measurements by Rasmussen [10]. He compared the measured and calculated acoustic transfer impedance of a coupler fitted with standard laboratory microphones and two narrow, open-ended tubes. The transfer impedance was measured using a reciprocity technique. Both tubes had a radius of 0.234 mm and a length of 1 m. Rasmussen reported a plot of the change (in dB) of the coupler impedance when the tubes were blocked compared to when the tubes were open. Rasmussen showed that a particular expression for the low-frequency acoustic impedance of a narrow tube (used to correct the coupler impedance for microphone calibrations) was subject to large systematic effects at sufficiently low frequency. Unfortunately, insufficient information was given to directly compare the measured and calculated impedance of a single tube without the coupler.

## 5. Discussion and Recommendations

**Fig. 17:**
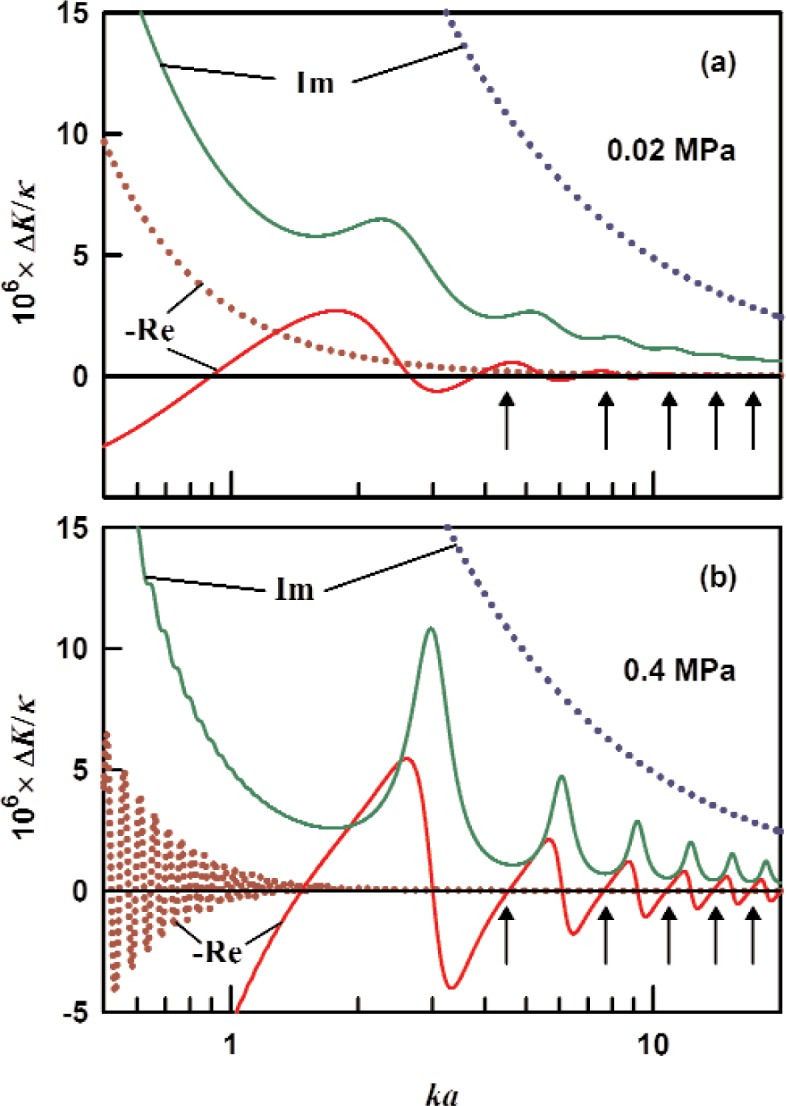
The perturbations for a spherical resonator from Eq. (9) for the single-stage duct (dotted) and two-stage duct (solid), discussed in Secs. 3.3 and 3.4, are plotted as a continuous function of *ka*. The sphere’s radius is assumed to be 5 cm. The values of ka for the radial modes are indicated by the arrows. The perturbations for a (5 cm radius) cylindrical resonator from Eq. (10) are obtained by multiplying the vertical scale by (0.2 m)/*L*.

Figure 17 shows Δ *K*/*κ* from Eq. (9) with *z*_0_*_n_* replaced by *ka* and ${\bar{y}}_{d}$ due to the single-stage duct (dotted) and two-stage duct (solid) considered in Secs. 3.3 and 3.4 plotted as a continuous function of *ka.* The predicted perturbations of the radial modes of a spherical cavity (with *a* = 5 cm) are found for the values of *ka* indicated by the arrows. The perturbations of the (0,2) mode from the single-stage duct are (0.20 + 10.7*i*)× 10^−6^ and (0.02 + 10.9*i*)× 10^−6^ at 0.02 MPa and 0.42 MPa, respectively. The perturbations of the (0,2) mode from the two-stage duct are (0.6 + 2.5*i*)× 10^−6^ and (−0.08 + 1.1*i*)× 10^−6^ at 0.02 MPa and 0.42 MPa, respectively. At the higher pressure (0.4 MPa), the modes of the sphere coincide with the anti-resonances of the short duct since we chose the length of the short duct *l*_d_ = *a*. The rapid oscillations that occur near and below *ka* = 1 are due to resonances in the 3 m long duct.

The perturbations for the longitudinal modes of a (5 cm radius) cylindrical cavity, from Eq. (10) with *lπ* replaced by *kL*, are obtained by multiplying the vertical axes in Fig. 17 by (0.2 m)/*L*. However, the 2-stage arrangement with *r*_d_ < *r*_d_′, used to generate the plots, is not optimal for a cylindrical cavity. For the quarter-wave duct, discussed in Sec. 3.2, with *r*_d_ = 0.7 mm, *r*_d_′ = 0.35 mm, *l*_d_ = 1.5*L*, and *L* = 2*R*, the perturbations of the two lowest even-symmetry modes (*kR* = *π* and 2*π*) at 0.02 MPa are (4.1 + 32.7*i*)× 10^−6^ and (0.03 + 16.2*i*)× 10^−6^; at 0.4 MPa the perturbations are (5.4 + 14.1*i*)× 10^−6^ and (3.5 + 18.4*i*)× 10^−6^, respectively. With this arrangement, two ducts are necessary to flow the test gas through the resonator. We recommend using two cylindrical resonators, one twice as long as the other, to take advantage of the cancellation of the end-plate perturbations at the same frequency. For this reason, the ducts for flowing gas and for remote transducers, if needed, should be placed on the resonator’s end plates.

To improve the experimental measurements of the admittance of small-diameter ducts, we recommend the following improvements in decreasing order of importance: 1) controlling the temperature and static pressure of the gas medium to reduce the random noise in the acoustic pressure, 2) devising a method to remove and reattach the tube such that the admittance is reproducible, so that the tube length can be determined more accurately and the coupler’s impedance can be measured directly, 3) using argon instead of air as the gas medium to reduce the uncertainty in the gas properties, and 4) blocking the end of the tube opposite the coupler or using another coupler to better define the terminal impedance.

We have examined the perturbations of the modes of an acoustic thermometer caused by circular ducts used either for gas flow or as acoustic waveguides coupled to remote transducers. We calculated the acoustic admittance of circular ducts using a model based on transmission line theory. We used the admittance to calculate the perturbations to the resonance frequencies and half-widths of the modes of spherical and cylindrical acoustic resonators as functions of the duct’s radius, length, and the locations of the transducers along the duct’s length. We measured the specific acoustic admittances of a series of ducts between 200 Hz and 10 kHz in ambient air and compared the results with theory. For a 1.4 mm inside-diameter, 1.4 m long tube, the root mean square difference between the measured and modeled specific admittances over this frequency range was 0.018 (the magnitude of the specific admittance was nominally one).

## Figures and Tables

**Fig. A1:**
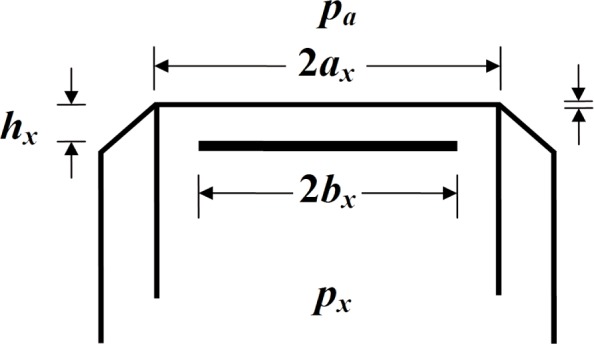
Sketch of B & K 4135 microphone cartridge.

## Footnotes

2 Certain commercial equipment, instruments, or materials are identified in this paper to foster understanding. Such identification does not imply recommendation or endorsement by the National Institute of Standards and Technology, nor does it imply that the materials or equipment identified are necessarily the best available for the purpose.

## 6. Appendix A. Condenser Microphone as a Source

A schematic diagram of the Bruel & Kjaer 4135 condenser microphone is shown in Fig. A1. The properties of the 4135 are listed in Table A1. The membrane has a thickness *t_x_* and is stretched in tension over an annular insulator (quartz). The membrane flexes with an effective radius *a_x_*. The gap *h_x_* separates the membrane from a backplate with radius *b_x_*. The membrane and the backplate form the two electrodes of a parallel-plate capacitor with capacitance $C_{x}=\varepsilon_{0}\pi b_{x}^{2}/h_{x}$. When used as a detector, the microphone is biased with a DC voltage of 200 V, and the charging current resulting from motion of the membrane is detected. However, when a condenser microphone is used as a source with sinusoidal drive and no DC bias, the sound waveform it generates is a sinusoid whose frequency is twice the excitation frequency. An advantage to this (2*f*) mode of operation is that cross talk between the high voltage excitation and the low voltage of nearby detectors is virtually eliminated.

**Table A1:** Properties of B & K 4135 microphone cartridge used a sound source

Property	Source	Symbol	Value	Unit
membrane radius	measured	*a*_x_	2.13	mm
membrane thickness	measured	*t*_x_	2.54	m
backplate radius	measured	b_x_	1.74	mm
gap	calculated	*h*_x_	13.0	m
membrane mass per unit area	calculated	*σ_M_*	0.0223	kg/m^2^
capacitance	manufacturer	*C*_x_	6.4	pF
membrane compliance	manufacturer	χ	1.5×10^−10^	m/Pa
microphone equivalent volume	manufacturer	*V*_x_	0.6	mm^3^

A very detailed model of condenser microphones is given in Ref. [21], but is beyond the scope of this article. Our objective in this appendix is to describe the low-frequency behavior of a condenser microphone used as a source. We solve a simplified equation of motion for a membrane under tension whose displacement from equilibrium is *y*. Additional forces are included to account for the reaction of the gas in the microphone volume and the surrounding environment. Thermoacoustic dissipation is neglected.

If we define *y* as positive for upward displacement, then a simplified equation of motion is

$$\sigma_{M}\frac{\partial^{2}y}{\partial t^{2}}=T\nabla^{2}y+p_{E}-\Delta p\;.$$ (A1)

The first term on the right hand side of Eq. (A1) is the restoring force per unit area due to the membrane tension, *p_E_* is the force per unit area from the applied electric field *E*

$$p_{E}=-\frac{1}{2}\varepsilon_{0}E^{2}=-\frac{\varepsilon_{0}}{2h_{x}^{2}}V^{2}\;,$$ (A2)

and Δ*p* = *p*_a_ − *p*_x_, where *p*_a_ and *p*_x_ are the acoustic pressures generated in front of and behind the membrane, respectively. The applied voltage $V\left(t\right)\;=V_{s}\;e^{i\omega_{s}t}$ is at frequency *f*_s_, but the electric force on the membrane oscillates at 2*f*_s_ and has a DC component. Since we are only concerned with the AC response, we replace *V*^2^ in Eq. (A2) by $V_{\text{rms}}^{2}\;e^{i\omega t}$ with *ω* = 2*ω*_s_. The steady-state response of the driven membrane displacement *y* (*t*) is also proportional to *e^iωt^*.

The source strength is defined as the rate of volume displacement averaged over the membrane area

$$U_{0}=\pi a_{x}^{2}\langle \dot{y}\rangle =i\omega \pi \;a_{x}^{2}\langle y\rangle \;.$$ (A3)

If the acoustic impedance of the medium in front the transducer is *Z_L_* and the impedance of the medium in the space inside the transducer is *Z*_x_, then we have

$$p_{a}=Z_{L}U_{0}$$ (A4)

and

$$p_{x}=-Z_{x}U_{0}\;.$$ (A5)

We rewrite Eq. (A1) as

$$\nabla^{2}y+K^{2}y=\frac{\varepsilon_{0}\;V_{\text{rms}}^{2}e^{i\omega t}}{2h_{X}^{2}T}+\frac{i\omega \pi a_{x}^{2}}{T}\left(Z_{L}+Z_{X}\right)\langle y\rangle$$ (A6)

where *K*^2^ = *σ_M_ω*^2^/*T*. The steady-state solution for Eq. (A6) can be expanded in terms of the normal modes of a circular membrane with a fixed edge, namely *J_m_*(*ξ_mn_r*/*a*_x_) cos (*mθ*), where *J_m_*(*ξ_mn_*) = 0. Due to the axial symmetry of the drive force, however, only the axially symmetric modes (*m* = 0) will be considered in the model, i.e.,

$$y=\sum_{n=1}^{\infty }A_{0n}J_{0}\left(\xi_{0n}r/a_{x}\right)e^{i\omega t}\;.$$ (A7)

The coefficients *A*_0_*_n_* are obtained by substituting (A7) into (A6) and integrating over the membrane area. For simplicity, we assume the membrane and backplate have the same diameters.

$$A_{0n}=-\frac{2a_{x}^{2}\left(-p_{E}+\Delta p\right)}{T\left(\xi_{0n}^{2}-K^{2}a_{x}^{2}\right)\xi_{0n}J_{1}\left(\xi_{0n}\right)}$$ (A8)

Equation (A8) predicts the lowest mode of the membrane occurs when *Ka*_x_ = *ξ*_01_ ≈ 2.4048 or about 74 kHz. The average membrane displacement in the low-frequency limit *Ka*_x_ ≪ *ξ*_01_ is

$$\langle y\rangle =\frac{-\varepsilon_{0}V_{\text{rms}}^{2}\chi /\left(2h_{x}^{2}\right)}{1+i\omega \pi a_{x}^{2}\left(Z_{c}+Z_{x}\right)\chi }$$ (A9)

where we have used the property $\sum_{n=1}^{\infty }\left(1/\xi_{0n}^{2}\right)=\frac{1}{32}$ and identified the membrane compliance $\chi =a_{x}^{2}/(8T)$. We can estimate *Z_L_* and *Z*_x_ assuming they represent simple volumes, *V_L_* and *V*_x_, respectively, with the expressions *Z_L_* = *pc*^2^/(*iωV_L_*) and *Z*_x_ = *pc*^2^/(*iωV*_x_). For our application, the volume of the coupler (134 mm^3^) is very large compared to the transducer’s equivalent volume (0.6 mm^3^), so we can neglect the impedance *Z_L_*. This is important because it means that the source strength will not depend strongly on the coupler or the attached tubes.

Finally, the source strength from Eq. (A3) in the low-frequency limit is (neglecting *Z_L_*)

$$U_{0}=\frac{-i\omega \pi a_{x}^{2}\;V_{\text{rms}}^{2}\;\varepsilon_{0}\chi /\left(2h_{x}^{2}\right)}{1+\chi \pi a_{x}^{2}\rho c^{2}/V_{x}}.$$ (A10)

In ambient air (*ρc*^2^ = 1.41 × 10^5^ Pa) at 1 kHz, Eq. (A10) gives

$$|U_{0}|\approx \left(0.091{\text{mm}}^{3}/s\right),$$ (A11)

using values from Table A1.

## References

[1] Michael R (1989). Moldover Acoustic and microwave resonances applied to measuring the gas constant and the thermodynamic temperature. IEEE Transactions on Instrumentation and Measurement.

[2] Moldover MR, Boyes SJ, Meyer CW, Goodwin ARH (1999). Thermodynamic temperatures of the triple points of mercury and gallium and in the interval 217 K to 303 K. J Res Natl Inst Stand Technol.

[3] Ripple DC, Defibaugh DR, Moldover MR, Strouse GF, Ripple DC (2003). Techniques for primary acoustic thermometry to 800 K, in Temperature: Its Measurement and Control in Science and Technology.

[4] Strouse GF, Defibaugh DR, Moldover MR, Ripple DC, Ripple DC (2003). Progress in primary acoustic thermometry at NIST: 273 K to 505 K, in Temperature: Its Measurement and Control in Science and Technology.

[5] Ripple DC, Strouse GF, Moldover MR (2007). Acoustic thermometry results from 271 K to 552 K. Int J Thermophys.

[6] Mehl JB, Moldover MR, Pitre L (2004). Designing quasi-spherical resonators for acoustic thermometry. Metrologia.

[7] Benedetto G, Gavioso RM, Spagnolo R, Marcarino P, Merlone A (2004). Acoustic measurements of the thermodynamic temperature between the triple point of mercury and 380 K. Metrologia.

[8] Gillis KA, Moldover MR, Goodwin ARH (1991). Accurate measurements in gases under difficult conditions. Rev Sci Instrum.

[9] Ripple DR

[10] Rasmussen K (1982). Note on the Acoustic Impedance of Narrow Tubes. Acoustica.

[11] Rodrigues D, Guianvarc’h C, Durocher JN, Bruneau M, Bruneau AM (2008). A method to measure and interpret input impedance of small acoustic components. J Sound and Vib.

[12] Gillis KA, Mehl JB, Moldover MR (2003). Theory of the Greenspan Viscometer. J Acoust Soc Am.

[13] Crandall IB (1927). Theory of vibrating systems and sound.

[14] Quinn TJ, Colclough AR, Chandler TRD (1976). A new determination of the gas constant by an acoustical method, Philosophical Transactions of the Royal Society of London. Series A: Mathematical and Physical Sciences.

[15] Morse PM, Ingard KU (1968). Theoretical Acoustics.

[16] Gillis KA, Moldover MR Designing ducts for acoustic thermometers.

[b17-v114.n05.a02] 17NIST Reference Fluid Thermodynamic and Transport Properties Database (REFPROP): Version 8.0

[18] Berg RF (2005). Simple flow meter and viscometer of high accuracy for gases. Metrologia.

[19] Daniels FB (1947). Acoustic impedance of enclosures. J Acoust Soc Am.

[20] Levine H, Schwinger J (1948). On the radiation of sound from an unflanged circular pipe. Phys Rev.

[21] Zuckerwar AJ, Wong GSK, Embleton TEW (1995). Principles of Operation of Condenser Microphones, in AIP Handbook of Condenser Microphones.

